# Supplementary material for: Smart Chip Technology for the Control and Management of Invasive Plant Species: A Review
Source: Plants (Basel). 2025 May 18;14(10):1510. doi: 10.3390/plants14101510 (PMC12114904; doi:10.3390/plants14101510)
Supplement: Supplementary file 1 [file plants-14-01510-s001.zip › plants-3565868-supplementary.pdf]

Table S1. The details of the smart sensor and chip with model's number used in precision agriculture and weed control

Sensor/Chip Model	Application Area	Functionality	Data Collection Method	Studied Crop/Weed	Source/Link
Sentera NDVI Sensor	Weed mapping, crop stress detection	Captures NDVI imagery for vegetation health	Drone-mounted multispectral imaging	Corn, soybean	https://sentera.com
SlantRange 4P + Sensor	Weed and pest detection	Multispectral + computer vision AI	Drone-based analysis	Broadacre crops	https://www.slantrange.com
Plantower PMS7003	Soil/air quality monitoring	Particulate concentration sensor for soil air pollution	Integrated into IoT & AI systems	Environmental monitoring	http://www.plantower.com/en/content/?107.html
CropX Soil Sensor	Soil moisture/nutrients	Measures EC, temp, moisture at various depths	Cellular-enabled, cloud-based	All major crops	https://www.cropx.com
Bosch BME688	VOCs and greenhouse gases	Gas sensor for air quality, compost maturity	Embedded in environmental sensor nodes	Soil compost and emissions	https://www.bosch-sensortec.com/products/environmental-sensors/gas-sensors/bme688/
BlueRiver See and Spray™	Herbicide application	Computer vision-based weed detection & spraying	Real-time onboard AI processing	*Amaranthus* spp. (waterhemp, *Palmer amaranth*)	https://bluerivertechnology.com/see-and-spray
Arable Mark 3	Microclimate + crop health	Combines weather, solar, and spectral sensors	Field-mounted with cloud platform	Vineyards, grains, legumes	https://www.arable.com/product
Teralytic Sensor	Soil nutrient profiling	Measures NPK, pH, temp, salinity	Underground probe with LTE/LoRa	Corn, soybean	https://www.teralytic.com
